# Prediction of individual immune responsiveness to a candidate vaccine by a systems vaccinology approach

**DOI:** 10.1186/1479-5876-12-11

**Published:** 2014-01-15

**Authors:** Annacarmen Petrizzo, Maria Tagliamonte, Maria Lina Tornesello, Franco M Buonaguro, Luigi Buonaguro

**Affiliations:** 1 Laboratory of Molecular Biology and Viral Oncology, Department of Experimental Oncology, Istituto Nazionale per lo Studio e la Cura dei Tumori, “Fondazione Pascale” - IRCCS, Naples, Italy

**Keywords:** Hepatitis C Virus, Non-Hodgkin’s Lymphoma, Idiotype vaccine, Immune response, Systems biology

## Abstract

**Background:**

We have previously shown that a candidate idiotype vaccine, based on the IGKV3-20 light chain protein, is able to induce activation of circulating antigen presenting cells (APCs) in both HCV-positive and HCV-negative subjects, with production of Th2-type cytokines. In addition, such a candidate idiotype vaccine induces an early gene expression pattern, characterized by the strong induction of an innate immune response, and a late pattern, characterized by a prevalent B cell response. Nonetheless, some HCV-positive individuals showed a complete lack of maturation of circulating APCs with low levels of cytokine production, strongly suggesting the possible identification of selective impairments in immune response in individual subjects.

**Method:**

Peripheral blood mononuclear cells (PBMCs) were stimulated *ex vivo* with IGKV3-20 for 24 h and 6 days. Analysis of the global gene expression profile as well as the cytokine pattern was performed for individual subjects.

**Results:**

The gene expression profile showed a strong agreement with the cytokine pattern. Indeed, the expression pattern of immune-related genes is highly predictive of the individual immunological phenotype.

**Conclusion:**

The overall results represent a proof of concept, indicating the efficacy of such an *ex vivo* screening platform for predicting individual’s responsiveness to an antigen as well as guiding optimization of vaccine design. Larger cohort study will be needed to validate results observed in the study.

## Introduction

Hepatitis C virus (HCV) is a Hepacivirus of the Flaviviridae family, mainly involved in hepatic disorders, including chronic hepatitis, cirrhosis and hepatocellular carcinoma (HCC) [1].

HCV has also been recognized as the major etiologic factor of type II mixed cryoglobulinemia (MC), an autoimmune disease ultimately leading to B cell non-Hodgkin’s lymphoma (NHL) in about 10% of MC patients [2-5].

The clonal B cell expansion is characterized by the production of an Ig molecule expressing a unique combination of antigen-specific sequences, the so called idiotype (Id). Therefore, the Id can be a suitable target for active, as well as passive immune-therapeutic strategies to eliminate the B cells driving the tumor [6,7].

In this respect, the IGKV3-20 idiotype has been selected as a potential target of either passive immune therapy or active vaccine strategy [8].

We have previously reported the results of the effect of the IGKV3-20 candidate idiotype vaccine on *ex vivo* stimulated PBMCs, as experimental platform for evaluation and prediction of responsiveness to vaccination [9]. IGKV3-20 light chain protein has been shown to induce activation of circulating APCs, i.e., CD14^+^ monocytes, as well as CD123^+^ plasmacytoid dendritic cells (pDCs) and CD11c^+^ myeloid DCs (mDCs), in both HCV-positive and HCV-negative healthy control subjects, with production of Th2-type cytokines [9,10]. No significant difference was observed between results obtained in human monocyte-derived dendritic cells (MDDCs) and circulating APCs, confirming previous results by us and other groups [11-15].

Moreover, such a candidate idiotype vaccine induces an early expression pattern, characterized by the induction of genes related to inflammatory response, and a late pattern, characterized by the induction of genes related to a B cell response [10].

Indeed, the Ingenuity Pathways Analysis (IPA) performed on “early” and “late” up-regulated genes showed a prevalence of inflammation and innate immunity-related pathways activated at 24 h post-induction, with significant overlapping between HCV-negative and positive groups, and a prevalence of “atypical” immune pathways activated at 6 days post-induction [10].

Nonetheless, some HCV-positive and negative subjects showed a poor response to the IGKV3-20 protein, with significant low levels of cytokine production and limited gene expression pattern.

In this regard, here we describe a systems biology approach to evaluate the individual responsiveness to the recombinant IGKV3-20 protein, aiming at identifying a possible impairment in the immune response and/or markers of responsiveness to such a specific antigen. Indeed, the specific effect of the recombinant IGKV3-20 protein has been evaluated *ex vivo* on human PBMCs of individual HCV-positive subjects via multiparametric analyses, including gene expression profiling combined to multiplex analysis of cytokines.

## Materials and methods

### Clinical specimens and cell treatment

Overall, samples from six HCV-positive subjects were analysed for the present study. Samples from five healthy donors were used as controls. Enrollment of subjects and treatment of derived human PBMCs have been previously described [10].

### Unsupervised analysis

For the unsupervised analysis a low-stringency filtering was applied, selecting the genes differentially expressed in 80% of all experiments with a >3 fold change ratio in at least one experiment. Hierarchical cluster analysis was conducted on the selected genes according to Eisen et al. [16]; differentially expressed genes were visualized by Treeview and displayed according to the central method [16,17].

### Supervised analysis

Supervised class comparison was performed using BRB ArrayTool developed at NCI, Biometric Research Branch, Division of Cancer Treatment and Diagnosis. Two subsets of genes were explored. The first subset included genes up-regulated in stimulated (IGKV3-20 treated) PBMCs compared to non-stimulated (PBS treated) PBMCs after 24 h incubation; the second subset included genes up-regulated in stimulated PBMCs compared to non-stimulated PBMCs after 6 days incubation.

Class comparison analyses were tested for an univariate significance threshold set at a *p-*value < 0.001. Gene clusters identified by the univariate *t*-test were challenged with two alternative additional tests, an univariate permutation test (PT) and a global multivariate PT.

Class comparison and hierarchical clustering were employed to determine the pattern of response and results are illustrated as a heat map of significance values. All analyses were performed using R and Cytoscape (http://www.cytoscape.org). Gene function was assigned based on Database for Annotation, Visualization and Integrated Discovery (DAVID) (http://david.abcc.ncifcrf.gov) and Gene Ontology (http://www.geneontology.org).

Ingenuity Pathways Analysis (IPA,http://www.ingenuity.com) was employed to elucidate the relationship and connection between differentially expressed genes.

## Results

### Cytokine pattern induced in circulating APCs

PBMCs obtained from enrolled subjects were incubated with IGKV3-20 for 24 h and 6 days and, at each time-point, levels of Th1 (IL-2 and TNF-α) and Th2 (IL-5, IL-6 and IL-10) cytokines were assessed by ELISA in the culture supernatant. The results show that the stimulation induces a significant production of both Th1 (TNF-α) and Th2 cytokines (IL-6 and IL-10) with a prevalent Th2-biased cytokine pattern, as previously reported [10].

**Figure 1:**
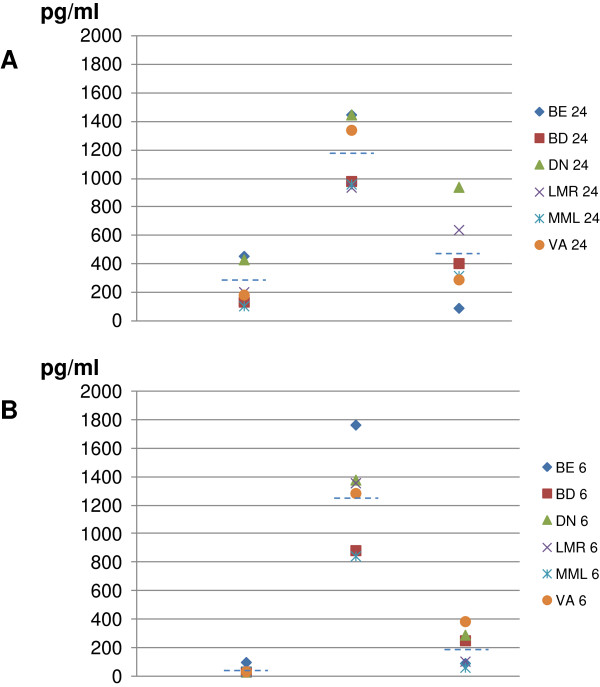
**Analysis of cytokine production in supernatant of PBMCs.** The cytokine profile induced by IGKV3-20 after 24 h **(A)** and 6d **(B)** incubation was evaluated in supernatant of stimulated PBMCs.

However, specific subjects show levels of cytokine induction significantly far from the mean value, indicating that differences may be observed in the response elicited by the antigen (Figure 1).

Indeed, at 24 h post-induction, PBMCs of subject DN always score at the top for release of TNF-α, IL-6 and IL-10 (1.7, 1.2 and 2.1 folds over the average, respectively). PBMCs of subject BE show a very similar pattern for TNF-α and IL-6 (1.8 and 1.2 folds over the average, respectively), whereas the amount of IL-10 released is the lowest (0.2 folds over the average). On the contrary, PBMCs of subjects MML and BD release the lowest levels of TNF-α and IL-6 (0.4 and 0.8 folds over the average, respectively) (Figure 1A).

Overall, high levels of IL-6 persist for the 6 days of induction, whereas the induction of IL-10 and, even more, TNF-α fades away at 6 days (Figure 1B).

Nevertheless, only PBMCs of subject BE (unlike those of DN) are confirmed to release the highest amount of TNF-α and IL-6, while PBMCs of subject VA release the highest amount of IL-10 (Figure 1B). As for the 24 h time-point, PBMCs of MML are confirmed to release the lowest amount of all three cytokines (Figure 1B). The overall results, therefore, indicate distinct cytokine patterns elicited by the same antigen *ex vivo* which may possibly reflect differences in individual response to the same antigen after *in vivo* vaccination.

### Identification of immune response pattern to IGKV3-20 at “early” time-point

Subsequently, the gene expression profile of samples from HCV-positive subjects, previously analyzed as whole group [10], was evaluated to identify individual patterns induced by recombinant IGKV3-20 on PBMCs from six HCV-positive subjects. To this aim, a supervised pair-wise comparison was performed between stimulated (IGKV3-20 treated) and non-stimulated (PBS) PBMCs.

The analysis at 24 h identified a clustering confirming the different response of samples BE (high) and MML (low) observed in the pattern of cytokine production induced by IGKV3-20 stimulation.

**Figure 2:**
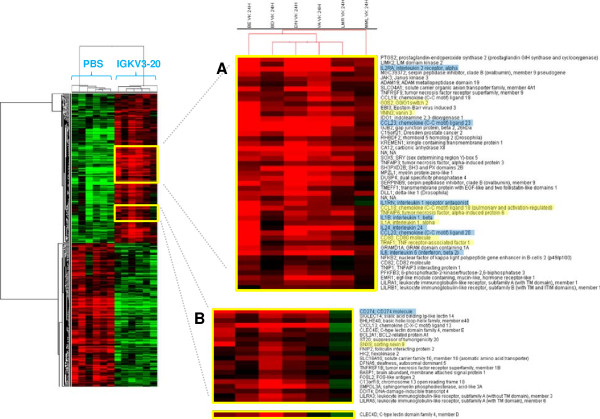
**Supervised analysis based on IGKV3-20 induction at 24 h.** Heat map of gene sets differentially modulated by IGKV3-20 after 24 h. The heat map in **(A)** shows several genes whose expression is strongly up-regulated in PBMCs of BE. The heat map in **(B)** shows several genes whose expression is down-regulated in PBMCs of MML after IGKV3-20 stimulation. Genes highlighted in yellow are mostly activated in sample BE. Genes highlighted in blue are activated in all HCV-positive samples, with exception of MML.

In particular, 394 genes differentially expressed were overall identified (201 up-regulated genes and 193 down-regulated genes) with the strongest gene activation induced in PBMCs of subject BE and the weakest one induced in PBMCs of subject MML (Figure 2). The remaining 4 samples showed an intermediate transcriptional pattern, suggesting the possible identification of high vs. weak responders according to results of simultaneous gene expression data. A list of modulated genes correlated with immunology functions is shown in Table 1.

**Table 1:** Immunology genes modulated by IGKV3-20 at 24 h

**Gene ID**	**Gene symbol**	**Gene name**	**Gene ID**	**Gene symbol**	**Gene name**
7960947	A2M	Alpha-2-macroglobulin	8154733	ACO1	Aconitase 1, soluble
8093294	CCR2	Chemokine receptor 2	8071899	ADORA2A	Adenosine A2a receptor
7937508	CD151	CD151 molecule	7990818	BCL2A1	BCL2-related protein A1
8133876	CD36	CD36 molecule	8089771	CD80	CD80 molecule
7953428	CD4	CD4 molecule	7898655	CDA	Cytidine deaminase
8115147	CD74	MHC, class II invariant chain	8042942	HK2	Hexokinase 2
8082035	CD86	CD86 molecule	8025601	ICAM1	Intercellular adhesion mol. 1
7966089	CMKLR1	Chemokine-like receptor 1	8097553	IL15	Interleukin 15
8115076	CSF1R	Colonystimul. factor 1 recep.	8054712	IL1A	Interleukin 1, alpha
8065403	CST3	Cystatin C	8054722	IL1B	Interleukin 1, beta
8166730	CYBB	Cytochrome b-245, beta polypeptide	8044574	IL1RN	Interleukin 1 receptor antagonist
8108370	EGR1	Early growth response 1	7931914	IL2RA	Interleukin 2 receptor, alpha
7985268	FAH	Fumarylacetoacetate hydrolase	8131803	IL6	Interleukin 6
816502	FBP1	Fructose-1,6-bisphosphatase 1	8077786	IRAK2	IL1 receptor-ass. kinase 2
8165011	FCN1	Ficolin 1	8035351	JAK3	Janus kinase 3
8058765	FN1	Fibronectin 1	7973336	MMP14	Matrix metallpeptidase 14
7913694	FUCA1	Fucosidase, alpha-L-1, tissue	7951217	MMP7	Matrix metallopeptidase 7
8129974	FUCA2	Fucosidase, alpha-L-2, plasma	8172220	NDP	Norrie disease
8163908	GGTA1	Glycoprotein, alpha-galactosyltransferase 1 pseudogene	7930074	NFKB2	Nuclear factor of kappa light polypeptide gene enhancer in B-cells 2 (p49/p100)
8180346	GPX1	Glutathione peroxidase 1	8075316	OSM	Oncostatin M
8157582	GSN	Gelsonlin	8062927	PI3	Peptidase inhibitor 3
7941936	GSTP1	Glutathione S-transferase pi 1	8150509	PLAT	Plasmin. activator, tissue
8140556	HGF	Hepatocyte growth factor	8037775	PTGIR	Prostagladin I2 receptor (IP)
8180086	HLA-DMA	MHC, class II, DM alpha	7922976	PTGS2	Prostag-endoperox synthase 2
8180078	HLA-DMB	MHC, class II, DM beta	7977786	SLC7A7	Solute carrier fam 7, memb 7
8180100	HLA-DPA1	MHC, class II, DP alpha 1	8130556	SOD2	Superoxide dismutase 2
8179519	HLA-DPB1	MHC, class II, DP beta 1	8066214	TGM2	Transglutaminase 2
8179481	HLA-DRA	MHC, class II, DR alpha	8045688	TNFAIP6	TNF, alpha-induced protein 6
8058552	IDH1	Isocitrate dehydrognase 1	7897877	TNFRSF1B	TNF receptor superfam, 1B
7942300	IL18BP	Interleukin 18 binding protein	7912145	TNFRSF9	TNF rec. superfam, memb 9
8070826	ITGB2	Intergrin, beta 2	8163825	TRAF1	TNF receptor-assoc. factor 1
8090162	ITGB5	Integrin, beta 5			
7957023	LYZ	Lysozyme			
8127854	ME1	Malic enzyme 1			
7926451	MRC1	Mannose receptor, C type 1			
8149448	MSR1	Macrophage scavenger recept 1			
8076403	NAGA	N-acetylgalactosaminidase, a			
8072744	NCF4	Neutrophil cytosolic factor 4			
8157650	PTGS1	Prostag-endoperoxsynthase 1			
7920271	S100A4	S100 calcium binding protein A4			
8136557	TBXAS1	Thromboxane A synthase 1			
8157524	TLR4	Toll-like receptor 4			
7924499	TLR5	Toll-like receptor 5			

Down-regulated (left) and up-regulated (right) genes.

The identified genes were further evaluated, to identify specific transcription profiles in the individual subjects. Considering the immune-related genes, heat maps in Figures 2A and B show a very strong up-regulation of specific genes mostly in sample BE (e.g., G0S2, VNN3, CCL18, TNFAIP6, IL-1A, CD80, TRAF1 and SNX9). An additional set of cytokine and cytokine receptor related genes, instead, shows a broad activation in all samples including BE (e.g., IL-1B, IL-6, IL-24, CCL23, CCL20 and IL2RA). The only exception is represented by the subject MML which shows a very limited pattern of gene activation and even down-regulation (Figure 2A and B).

Among the genes more strongly activated in BE, the TNF alpha-induced protein 6 (TNFAIP6), TNF receptor-associated factor 1 (TRAF1), G0/G1 switch gene 2 (G0S2), vanin-3 (VNN3) and sorting nexin 9 (SNX9) possibly suggest the activation of a TNF-mediated inflammatory pattern induced by IGKV3-20. Indeed, both TNFAIP6 and TRAF1 are downstream mediators of TNF signaling [18][19], whereas G0S2 is known to be induced by TNF through the activation of the NFKB complex [20]. Moreover, SNX9 promotes internalization of TNFR [21], whereas VNN3 induction is mediated by Th17/Th1 type cytokines, including TNF-α [22].

**Figure 3:**
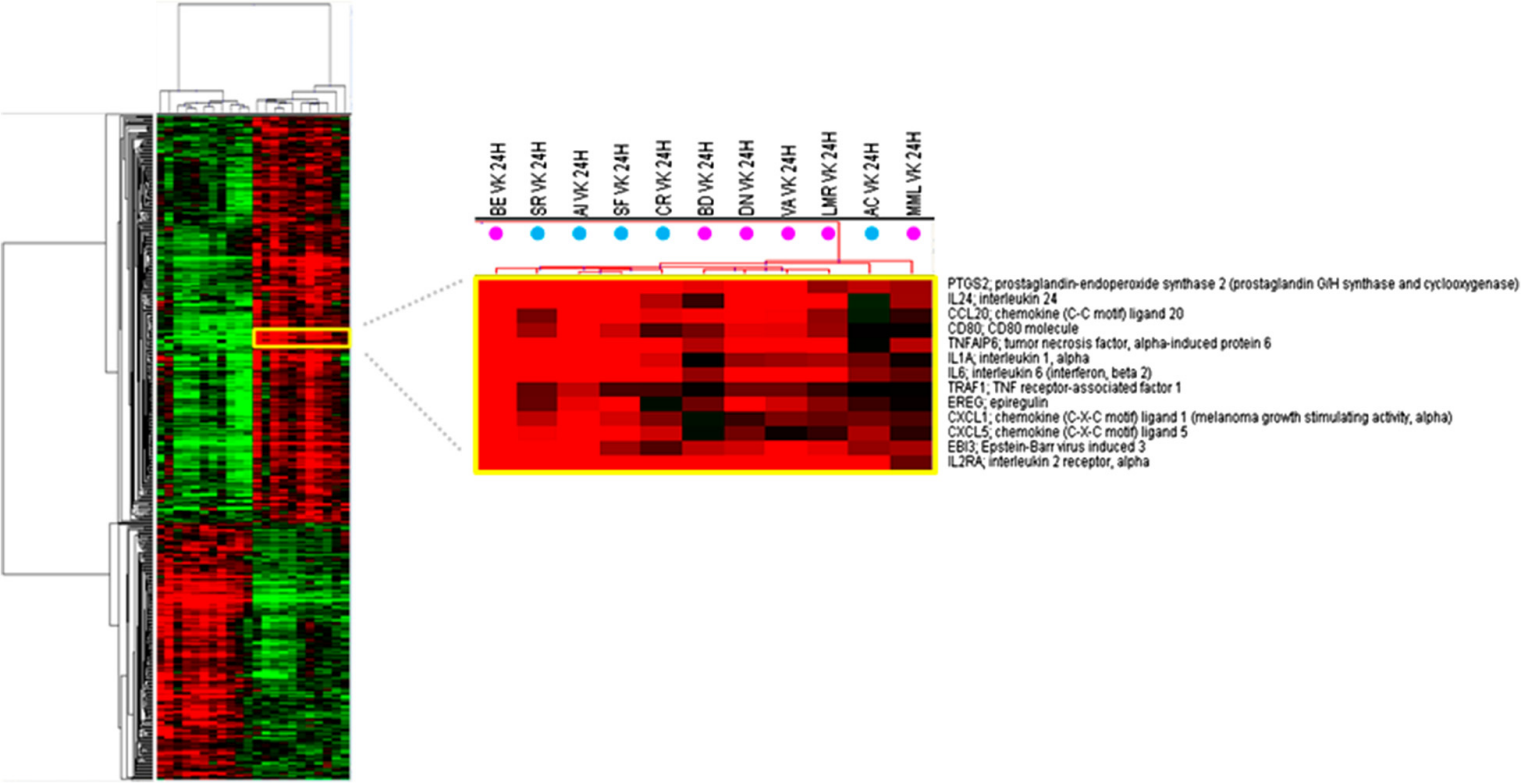
**Supervised analysis based on IGKV3-20 induction at 24 h.** Heat map of gene sets differentially modulated by IGKV3-20 after 24 h, in HCV-positive (pink dots) and HCV-negative healthy control (blue dots) subjects. The dendrogram at the top of the matrix indicates the degree of similarity between samples.

Indeed, the pronounced pro-inflammatory pattern induced by IGKV3-20 in PBMCs of BE is more similar to the expression profile elicited in samples from healthy control subjects (Figure 3).

Furthermore, the strong up-regulation of CCL18 and CD80 molecules confirms the significant activation of circulating APCs by IGKV3-20 (Figures 2A and 3) [10].

On the other hand, PBMCs of subject MML show down-regulation of tumor necrosis factor receptor superfamily member 1B (TNFRSF1B), a mediator of most of the metabolic effects of TNF-α, as well as of interleukin 1 receptor antagonist (IL1RN), whose expression is implicated in the modulation of the inflammasome (Figure 2A and B) [23]. This observation suggests the limited pro-inflammatory effect of TNF-α and IL-1 induced by IGKV3-20 in PBMCs of MML.

Furthermore, C-type lectin domain family 4 member D (CLEC4D), involved in antigen uptake for processing and further presentation to T cells, is strongly down-regulated in PBMCs of MML upon treatment with IGKV3-20, suggesting a possible impairment of the antigen uptake, processing and presentation pathway [24,25].

Unexpectedly, CD274 molecule, which has been speculated to play a major role in suppressing the immune system during autoimmune disease and disease states, including hepatitis, is down-regulated by IGKV3-20 in PBMCs of MML while strongly up-regulated in all the other HCV-positive samples (Figure 2B) [26].

### Identification of immune response pattern to IGKV3-20 at “late” time-point

**Figure 4:**
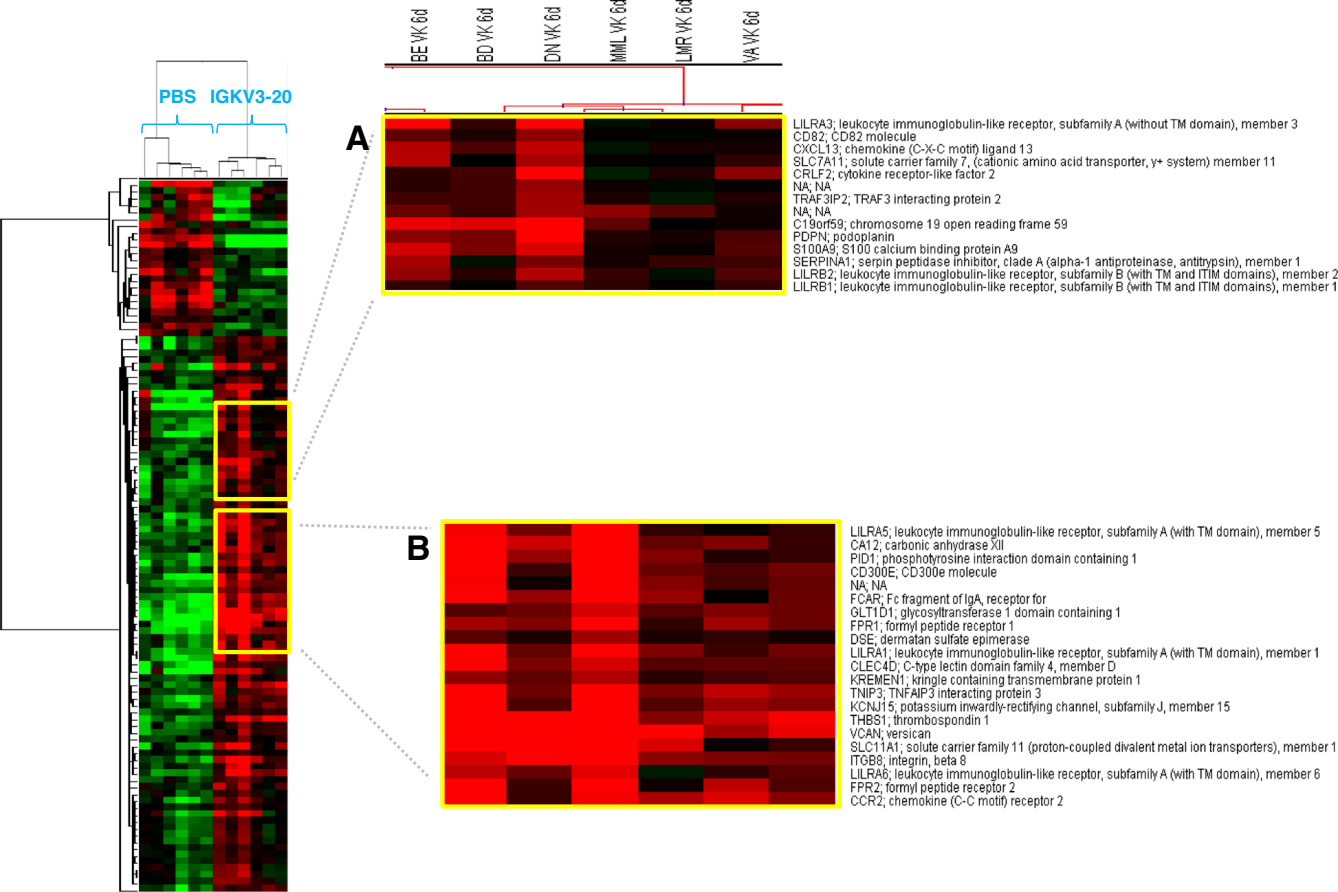
**Supervised analysis based on IGKV3-20 induction at 6d.** Heat map of gene sets differentially modulated by IGKV3-20 after 6 days. The heat map in **(A)** shows several genes whose expression is down-regulated in PBMCs of MML. The heat map in **(B)** shows several genes whose expression is strongly up-regulated in PBMCs of BE.

To evaluate a change in the gene expression pattern over-time, the transcriptional profiling analysis was performed also after 6 days (“late”) incubation (Figure 4).

The comparison analysis at this late time-point confirmed the different clustering of samples BE and MML. Overall, 105 genes differentially expressed were identified, of which 82 up-regulated and 23 down-regulated upon IGKV3-20 stimulation. A list of modulated genes correlated with immunology functions is shown in Table 2. The number of differentially expressed genes at late time-point is drastically reduced compared to the early time-point, but it is still indicative of a late modulation of the immune response by IGKV3-20.

**Table 2:** Immunology genes modulated by IGKV3-20 at 6 days

**Gene ID**	**Gene symbol**	**Gene name**	**Gene ID**	**Gene symbol**	**Gene name**
7960947	A2M	Alpha-2-macroglobulin	8093294	CCR2	Chemokine receptor 2
8127854	ME1	Malic enzyme 1, NADP(+)-dependent, cytosolic	8063386	CEBPB	CCAAT/enhancer binding protein (C/EBP), beta
7948444	TCN1	Transcobalamin I	8031374	FCAR	Fc fragment of IgA, receptor for
			7921873	FCGR3A	Fc fragment of IgG, low affinity
					Illa, receptor (CD16a)
			8038899	FPR1	Formly peptide receptor 1
			8042942	HK2	Hexokinase 2
			8047086	NAB1	NGFI-A binding protein 1 (EGR1 binding protein 1)
			7920244	S100A8	S100 calcium binding protein A8
			7982597	THBS1	Thrombospodin 1

Down-regulated (left) and up-regulated (right) genes.

Among the up-regulated genes CD300E, CLEC4D and FCAR are mostly activated in sample BE (Figure 4B). Moreover, several members of the leukocyte immunoglobulin-like receptor (LILR) family (i.e., LILRA1, LILRA3 and LILRA5) are activated, indicating a relevant role for such innate immune receptors, expressed on monocytes and B cells, in the response elicited by the IGKV3-20 (Figure 4A and B)[27].

**Figure 5:**
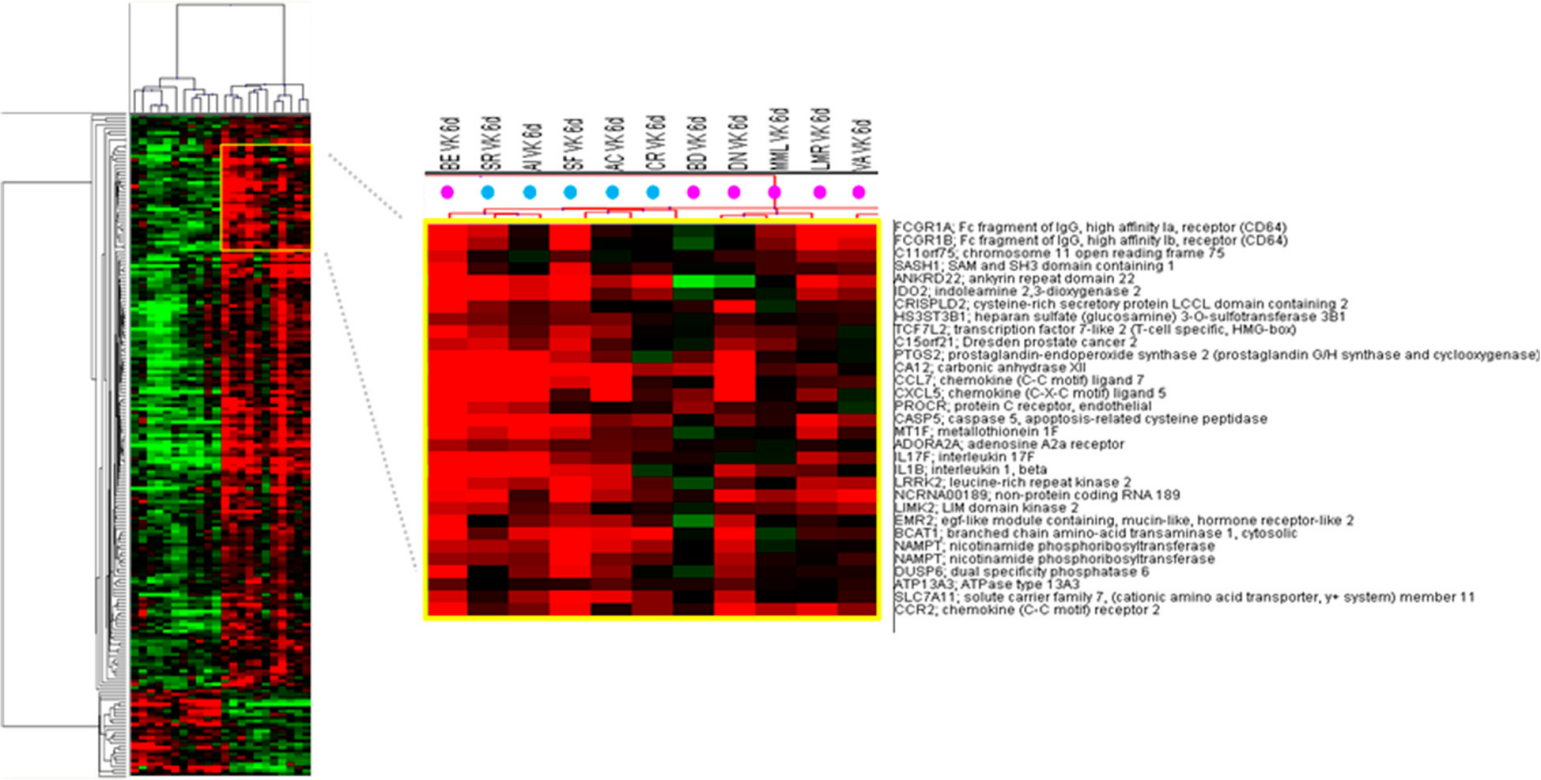
**Supervised analysis based on IGKV3-20 induction at 6d.** Heat map of gene sets differentially modulated by IGKV3-20 after 6 days in HCV-positive (pink dots) and HCV-negative healthy control (blue dots) subjects. The dendrogram at the top of the matrix indicates the degree of similarity between samples.

Even at 6 days the expression pattern induced by IGKV3-20 in PBMCs of BE strongly resembles the expression profile elicited by the same antigen in a cohort of healthy control subjects, as indicated by the dendrogram at the top of matrix in Figure 5.

On the other hand, immune genes such as LILRA3, CXCL13, CRLF2 and CD82 are even down-regulated in sample MML, suggesting the inefficient delivery of co-stimulatory signals for the T cell receptor (TCR)/CD3 pathway for such a subject (Figure 4A) [28].

### Identification of unique immune signatures in PBMCs of BE and MML

A subsequent supervised analysis was performed individually comparing sample BE or MML to all the other samples, and such analysis included all genes modulated by IGKV3-20 without subtracting the basal expression patterns (i.e., PBS).

In regards to PBMCs of subject BE, overall 102 differentially expressed genes were identified at 24 h post stimulation, of which 62 up-regulated and 40 down-regulated genes (data not shown).

**Figure 6:**
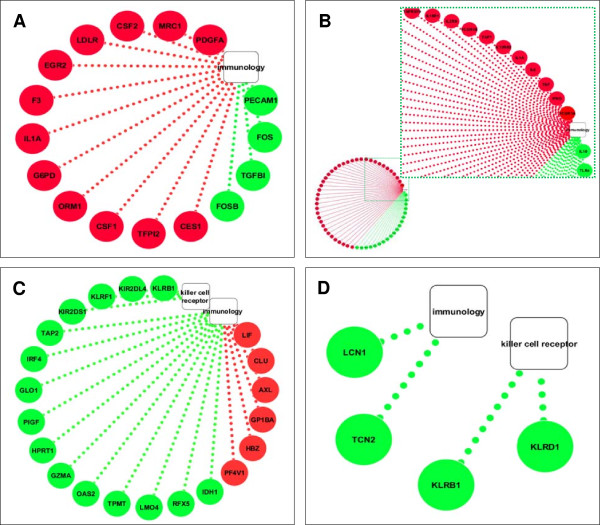
**Unique immune signatures induced in PBMCs of BE and MML.** Cytoscape analysis of immunology genes modulated by IGKV3-20 in PBMCs of BE at 24 h **(A)** and 6d **(B)**. Cytoscape analysis of immunology genes and killer cell receptor genes modulated by IGKV3-20 in PBMCs of MML at 24 h **(C)** and 6d **(D)**.

Among the up-regulated genes, several were identified as immunology genes and the resulting immunology network was visualized using Cytoscape (http://www.cytoscape.org) (Figure 6A).

**Figure 7:**
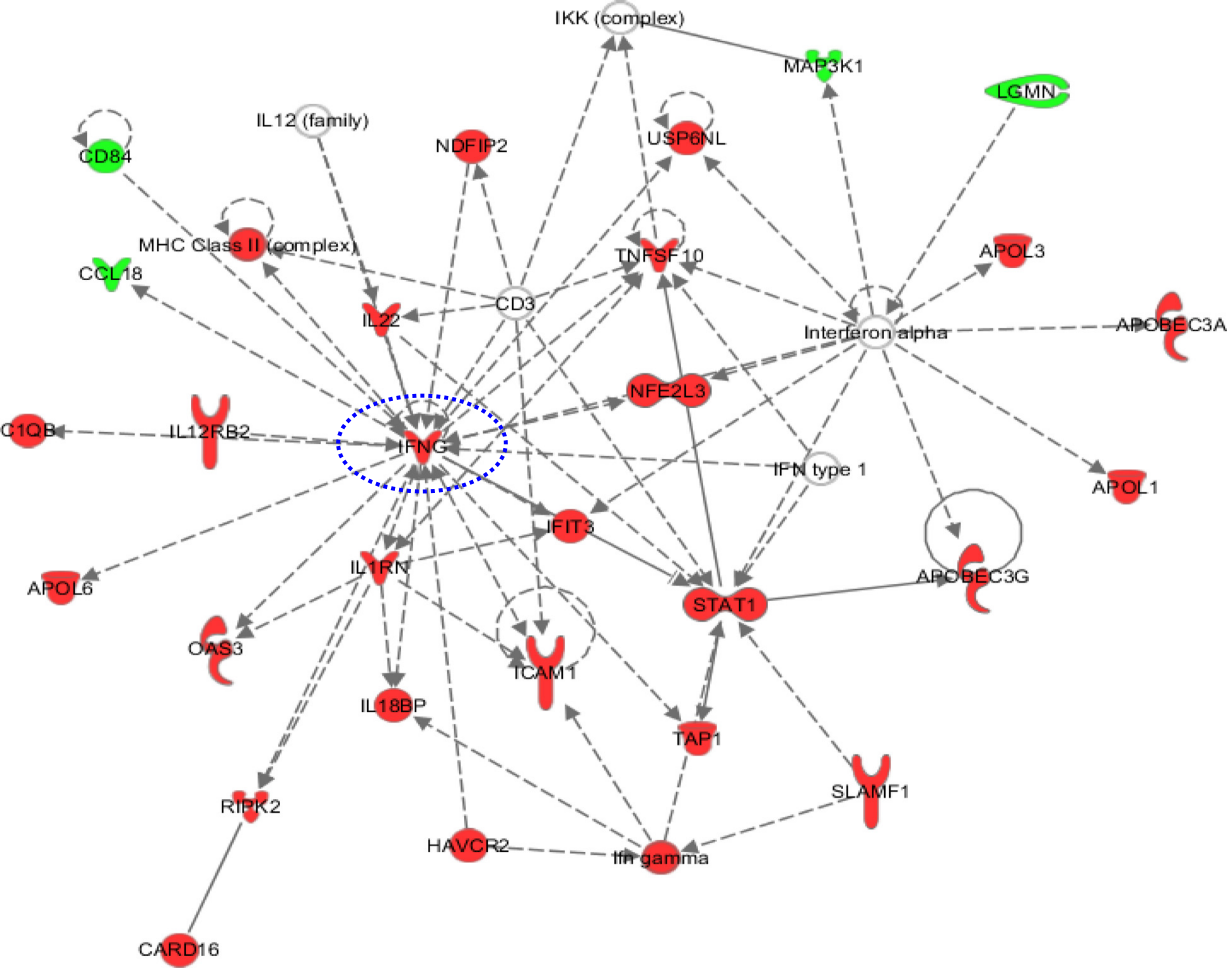
**Ingenuity pathways analysis.** IFNγ signaling pathway uniquely activated in PBMCs of subject BE after 6 days stimulation with IGKV3-20.

At 6 days post stimulation, overall 617 differentially expressed genes were identified, of which 349 up-regulated and 268 down-regulated genes (data not shown). Interestingly, a unique gene signature was identified, characterized by the up-regulation of Th1 cytokine (i.e., TNF and IFNγ) and cytokine receptor (i.e., IL2RB, IL18R1 and IL12RB2) genes, as well as high-affinity Fc-gamma receptor genes (i.e., FCGR1A and FCGR1B) (Figure 6B). All these genes contribute to the inflammatory response, promoting proliferation of natural killer (NK) cells, as well as T-cells of the Th1 phenotype, strongly suggesting a loop of specific activation of the IFNγ signaling [29], confirmed also by the IPA analysis (Figure 7).

On the other hand, in PBMCs of subject MML, the analysis identified overall 479 differentially expressed genes at 24 h, whereas 122 genes were differentially expressed at 6 days (data not shown). However, marker genes of the immune response were not identified up-regulated and most of the identified genes were not even annotated.

Interestingly, several immunology and killer-cell-receptor genes (i.e., KLRD1, KLRB1, KLRF1, KIR2DL4, KIR2DS1) were found down-regulated (Figure 6C and D), suggesting a possible impairment of the NK cell function in such a subject [30].

The overall results suggest the identification of molecular markers of individual response to a specific antigen, which could represent a platform for the identification of common predictive signatures of response to vaccination.

## Discussion

A major challenge in vaccinology is to predict vaccine efficacy [31-33]. Here, we used a multiparametric systems biology approach to identify gene signatures predictive of an immune response, using an experimental platform based on PBMCs from 6 HCV-positive subjects stimulated *ex vivo* with the IGKV3-20 light-chain protein, as candidate idiotype vaccine.

The cytokine pattern induced by IGKV3-20 was assessed by ELISA in culture supernatant of stimulated PBMCs, after 24 h or 6 days of incubation (Figure 1).

The results show that the stimulation induces an overall significant production of both Th1 (TNF-α) and Th2 cytokines (IL-6 and IL-10), with a prevalence of the latters. However, specific samples consistently show very different levels of TNF-α and IL-6 production, which are highest for samples BE and DN and lowest for sample MML. These results, although based on a small cohort, indicate a significant difference in the individual response to the same antigen and, in particular, suggest that subject BE is characterized by a relevant pro-inflammatory pattern with the highest levels of IL-6 and TNF-α and the lowest level of the anti-inflammatory IL-10 (Figure 1).

The global gene expression profile of PBMCs stimulated with IGKV3-20 for 24 h and 6 days confirmed such strong differences between BE and MML.

Indeed, in agreement with the cytokine expression pattern, the sample from subject BE shows the highest number of strongly activated genes, whereas the sample from subject MML shows the weakest transcriptional profile (Figures 2 and 4).

In particular, considering immune-related genes up-regulated at 24 h, the activation of a TNF-mediated inflammatory pattern in subject BE induced by IGKV3-20 is strongly suggested (Figure 2A). Moreover, the up-regulation of CCL18 and CD80 molecules suggests a significant activation of circulating APCs in this subject (Figure 2A). On the contrary, several immune-related genes are even down-regulated in PBMCs of MML, suggesting a possible impaired activation of a pro-inflammatory and/or immune response by the antigen.

The strong difference in transcriptional profile of immune-related genes between subjects BE and MML is confirmed also after 6 days of incubation with IGKV3-20 (Figure 4).

Indeed, BE shows the gene activation of several members of the LILR (i.e., LILRA1, LILRA3 and LILRA5) family of immunoreceptors, as well as CD300E, CLEC4D and FCAR (Figure 4A and B).

More importantly, the supervised analysis performed on the pool of genes modulated by IGKV3-20, without subtracting PBS pattern, identified a late transcriptional profile characterized by the up-regulation of TNF, IFNγ, IL2RB, IL18R1, IL12RB2, FCGR1A and FCGR1B genes (Figure 6B), which is unique for subject BE and suggestive of a possible Th1-polarization of the immune response.

In this respect, the pronounced pro-inflammatory pattern induced by IGKV3-20 in PBMCs of BE strongly resembles the expression profile elicited by the same antigen in a cohort of HCV-negative healthy control subjects, as indicated in Figures 3 and 5.

On the contrary, a global down-regulation of immune genes such as killer cell receptor genes in PBMCs of MML may even suggest the inefficient establishment of NK cell-mediated innate immune response to the candidate idiotype vaccine.

Overall, the multiparametric analysis performed on PBMCs loaded *ex vivo* with the IGKV3-20 candidate idiotypic vaccine shows that the identification of specific gene transcriptional patterns to confirm differences in the immune response evaluated by means of different parameters (e.g., cytokine profile) is feasible.

Indeed, subjects BE and MML are clearly different regardless the parameters used to analyze the *ex vivo* effect of the IGKV3-20 on their PBMCs, suggesting a possible marked diversity of their responsiveness to such an antigen if administered *in vivo*.

In conclusion, the present study represents a proof of concept and larger cohort studies will be needed to validate the results. Nevertheless, our results strongly suggest that our *ex vivo* screening platform is potentially useful to identify “proficient” prediction markers of individual responsiveness to a specific antigen, or classes of antigens (e.g., peptides, proteins, DNA), as well as to guide optimization of vaccine design.

Moreover, systems biology approaches not only allows the scrutiny of a global picture of vaccine-induced immune effect but can be also used to uncover new correlates of vaccine efficacy [34].

## Competing interests

The authors declare that they have no competing interests.

## Authors’ contributions

AP conducted the statistical analyses and wrote the paper; MT and MLT contributed to the statistical analyses; FMB and LB conceived and designed the study. All authors read and approved the final manuscript.
